# Toxicant-Induced Metabolic Alterations in Lipid and Amino Acid Pathways Are Predictive of Acute Liver Toxicity in Rats

**DOI:** 10.3390/ijms21218250

**Published:** 2020-11-04

**Authors:** Venkat R. Pannala, Shanea K. Estes, Mohsin Rahim, Irina Trenary, Tracy P. O’Brien, Chiyo Shiota, Richard L. Printz, Jaques Reifman, Masakazu Shiota, Jamey D. Young, Anders Wallqvist

**Affiliations:** 1 Department of Defense Biotechnology High Performance Computing Software Applications Institute, Telemedicine and Advanced Technology Research Center, U.S. Army Medical Research and Development Command, Fort Detrick, MD 21702, USA; jaques.reifman.civ@mail.mil; 2 The Henry M. Jackson Foundation for the Advancement of Military Medicine, Inc., Bethesda, MD 20817, USA; 3 Department of Molecular Physiology and Biophysics, Vanderbilt University School of Medicine, Nashville, TN 37232, USA; shanea.estes@vanderbilt.edu (S.K.E.); tracy.obrien@vanderbilt.edu (T.P.O.); chiyo.shiota@vanderbilt.edu (C.S.); Richard.printz@vanderbilt.edu (R.L.P.); masakazu.shiota@vanderbilt.edu (M.S.); 4 Department of Chemical and Biomolecular Engineering, Vanderbilt University School of Engineering, Nashville, TN 37232, USA; mohsin.rahim@vanderbilt.edu (M.R.); irina.trenary@Vanderbilt.Edu (I.T.)

**Keywords:** liver, genome-scale metabolic models, mechanism, hepatotoxicity, injury pathways, gene expression profiles, and metabolomics

## Abstract

Liver disease and disorders associated with aberrant hepatocyte metabolism can be initiated via drug and environmental toxicant exposures. In this study, we tested the hypothesis that gene and metabolic profiling can reveal commonalities in liver response to different toxicants and provide the capability to identify early signatures of acute liver toxicity. We used Sprague Dawley rats and three classical hepatotoxicants: acetaminophen (2 g/kg), bromobenzene (0.4 g/kg), and carbon tetrachloride (0.3 g/kg), to identify early perturbations in liver metabolism after a single acute exposure dose. We measured changes in liver genes and plasma metabolites at two time points (5 and 10 h) and used genome-scale metabolic models to identify commonalities in liver responses across the three toxicants. We found strong correlations for gene and metabolic profiles between the toxicants, indicative of similarities in the liver response to toxicity. We identified several injury-specific pathways in lipid and amino acid metabolism that changed similarly across the three toxicants. Our findings suggest that several plasma metabolites in lipid and amino acid metabolism are strongly associated with the progression of liver toxicity, and as such, could be targeted and clinically assessed for their potential as early predictors of acute liver toxicity.

## 1. Introduction

The liver plays a central role in a wide range of physiological functions, including metabolism, distribution of nutrients, and detoxification of toxic metabolites and ingested substances. The liver is a metabolically active organ that utilizes carbohydrates for synthesis of cholesterol as well as fatty acids for the production of triglycerides for export and storage. It also plays a critical role in maintaining blood glucose levels during fasting by synthesis of glucose from lactate and amino acids, and it converts excess fatty acids into ketone bodies for energy under prolonged starvation. Furthermore, the liver exports fatty acids as triglycerides to adipose tissue for storage and to muscle and other tissues for energy metabolism. Thus, the liver plays a major role in overall human metabolism, and any impairments in the liver metabolic processes due to external factors can lead to liver disorders ranging from simple cellular necrosis to complicated diseases, such as non-alcoholic fatty liver disease, fibrosis, cirrhosis, and liver cancer, which are serious threats to public health [1,2,3,4].

Several internal and external factors, such as genetic background, gut microbiota, and dietary factors, act simultaneously in the initiation and progression of liver diseases [5,6]. In addition, recent studies have suggested a link between drug and environmental chemical exposure and the initiation and progression of several liver disease processes [7,8,9]. The liver is the major organ for the metabolism of drugs and environmental chemicals, wherein an acute or chronic exposure of some of these agents may lead to lipid accumulation, mitochondrial dysfunction, modulation of nuclear receptor activation, and oxidative stress [10,11,12]. For example, chronic treatment with amiodarone, an anti-arrhythmic drug, produces steatosis in patients through impaired mitochondrial function and inhibition of lipid oxidation [13]. Similarly, acute bromobenzene exposure in rats induces steatosis with lipid accumulation in the liver [14]. Indeed, several drugs and environmental chemicals, such as acetaminophen, carbon tetrachloride, and thioacetamide, are in regular use as model liver toxicants to induce a specific liver disease phenotype and understand their pathophysiological mechanisms [15,16,17].

Even though it is evident that toxicant-induced disturbances in hepatocyte metabolism, such as lipid accumulation, mitochondrial disruptions, and oxidative stress, are hallmarks of liver disease processes [10,11,12], the underlying mechanisms leading to these alterations remain elusive. Therefore, it is difficult to track the very early onset and initiation of disease, events that are key for the early diagnosis and effective therapeutic interventions to prevent injury. Deciphering the underlying mechanisms leading to pathophysiological processes in liver metabolism will ultimately help us prevent disease progression and incipient liver failure. To advance a detailed understanding of these mechanisms, we require knowledge of the coordinated behavior of a large number of interconnected metabolic processes and a systematic approach to link these events to injury-specific alterations. Advancements in large-scale high-throughput technologies, such as RNA-sequencing (transcriptomics) and global plasma metabolic profiling studies (metabolomics), provide an opportunity to detect subtle disturbances caused by a toxicant exposure on liver metabolism. We hypothesize that such changes in gene expression patterns and metabolic profiles can be predictive of toxicity, despite differences in mechanism of action, if they reflect perturbations in vital cellular pathways that remain common to multiple liver toxicants. Furthermore, employment of a systems biology approach capable of integrating genomic, transcriptomic, and metabolomic data can extend our understanding of these molecular mechanisms, which in turn enables identification of potential indicators for liver damage [18,19,20,21,22].

In this study, we explored the combination of transcriptomics and metabolomics integrated with genome-scale models to acquire a comprehensive perspective on the similarities in rat liver metabolism exposed to three model hepatotoxicants. We chose rats, which share similarities with humans in terms of metabolism [18], and knowledge obtained from rats has shown a potential for being translated to human studies [23]. We selected the routinely used hepatotoxicants acetaminophen (APAP), bromobenzene (BB), and carbon tetrachloride (CCl_4_) based on their well-known hepatotoxicity and measured changes in liver metabolism using RNA-sequencing, global plasma metabolic profiling, and ^2^H/^13^C metabolic flux analysis (MFA). To capture system-level metabolic changes, we subjected male Sprague Dawley rats to a single acute dose of one of these three model toxicants and included the appropriate control groups for the acquisition of liver gene expression profiles and plasma metabolic profiles 5 and 10 h after toxicant exposure under identical conditions. Furthermore, under similar short-term fasting conditions, we also measured changes in absolute hepatic fluxes in central carbon metabolism to decipher similarities in liver response to these different toxicants. These combined experiments allowed us to determine early changes in the liver metabolism before substantial cellular damage occurs and provided an opportunity to identify common signatures of liver toxicity upon exposure to different toxicants.

Our global analysis revealed that changes in liver gene expression and plasma metabolic profiles were highly correlated between APAP and BB, indicating a similar progression of toxicity with only minor differences in hepatic fluxes related to glucose production. Our study captured the well-known acute response of CCl_4_ [24], with peak perturbations to liver metabolism occurring within 5 h after exposure. Furthermore, our analysis showed that multiple gene and metabolic changes at the peak response for CCl_4_ (5 h after exposure) were highly correlated with the peak responses observed at 10 h after APAP or BB exposure, suggesting commonalities in the liver response to all three toxicants. Our integrated analysis further highlighted common changes in liver metabolism and identified several injury-specific pathways in lipid, amino acid, and carbohydrate metabolism that responded similarly to these hepatotoxicants. Interestingly, our model-based analysis suggested that several metabolites in lipid metabolism were strongly associated with changes in liver gene expression across the three toxicants, which could serve as early-stage metabolite signatures of liver toxicity that can be assessed from plasma samples.

## 2. Results

**Figure 1:**
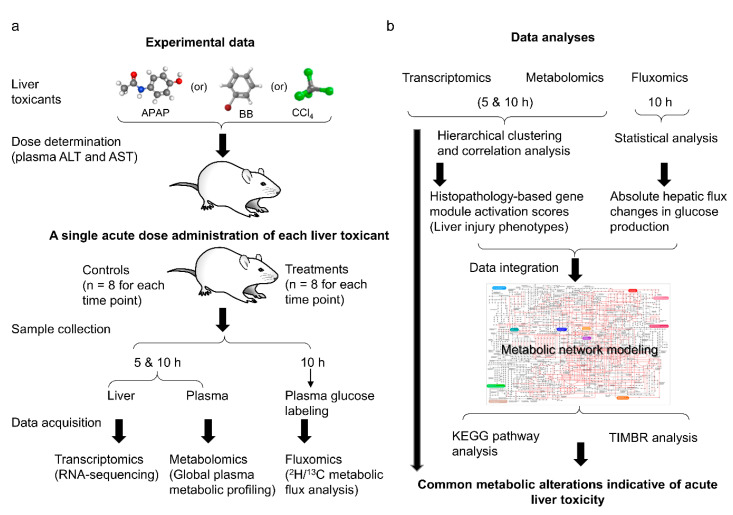
A schematic diagram showing the summary of experimental data generation and computational analyses. (**a**) Experimental study design to capture early perturbations in liver metabolism using transcriptomics, metabolomics, and fluxomics. (**b**) Global analysis of omics data to identify commonalities in liver response to toxicant exposure and data integration using genome-scale metabolic models to identify common metabolic signatures indicative of liver toxicity.

Figure 1 outlines the overall experimental design and computational analyses used in this study. To capture early changes in liver metabolism, we first performed dose-response studies for each toxicant separately (APAP, BB, or CCl_4_) and identified the lowest effective dose at which the standard liver biomarkers alanine aminotransferase (ALT) and aspartate aminotransferase (AST) indicated abnormalities in liver function (Supplementary Figure S1) [25,26]. Subsequently, we used this optimal acute dose to measure early perturbations in liver metabolism using three different data sources (Figure 1a). We used transcriptomics and metabolomics to measure changes in liver gene expression and plasma metabolite levels via RNA-sequencing and global metabolic profiling respectively, at two time points, one early (5 h) and another late (10 h). Additionally, we used in vivo isotope-labeled glucose infusion studies together with metabolic flux analysis to assess changes in absolute fluxes in the glucose production pathways of central carbon metabolism at the later time point (10 h). We then used statistical methods to analyze the significant changes in genes, metabolites, and fluxes in each study, and performed a global analysis using hierarchical clustering and liver injury module activation [27,28] to identify similarities across the toxicants (Figure 1b). Finally, to infer causality and identify mechanism-based similarities, we used a rat genome-scale metabolic model [29] and integrated changes in genes at the tissue level together with the measured hepatic fluxes as constraints to predict changes in plasma metabolite levels that could be compared against plasma metabolic profiling data (see Method Section for details). We describe the results of these analyses in the following sections.

### 2.1. Common Genes That Changed Significantly Are Highly Correlated between Toxicants

**Figure 2:**
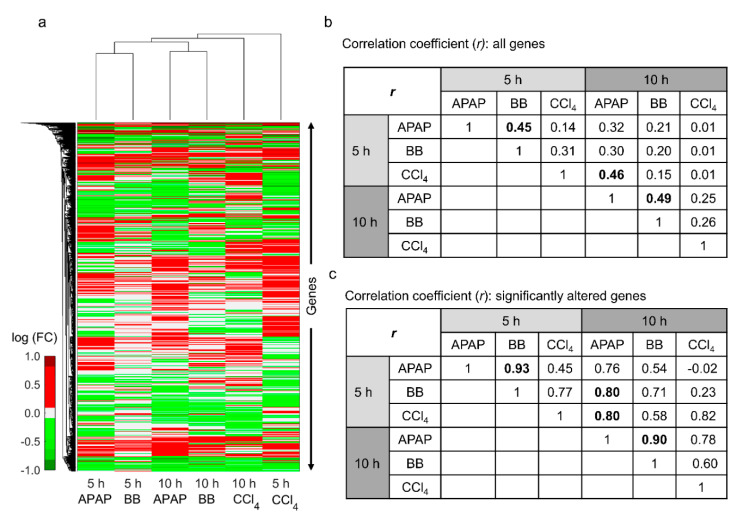
Identification of commonalities in liver gene expression changes from rats exposed to either APAP, BB, or CCl_4_. (**a**) Hierarchical clustering analysis of all common genes in the rat liver at 5 and 10 h after exposure of the toxicant. Red and green bars indicate up- and down-regulated genes, respectively; grey bars indicate unchanged genes. Pairwise correlation of the natural logarithmic fold changes (log(FC)) in genes common between the two toxicants (**b**) without using any significance cut-off values and (**c**) with using only significantly altered genes (false discovery rate (FDR) < 0.1). Pairwise combinations with high correlation coefficient values are indicated in bold.

Figure 2a shows the hierarchical clustering analysis of logarithmic fold change (log(FC)) values of each toxicant relative to their respective controls across all exposure time points. The results show that the liver gene expression profiles for APAP and BB were clustered together at both the early (5 h) and late (10 h) time points, indicating commonalities in liver response to these toxicants. However, changes in gene expression were clustered separately for the CCl_4_-administered study, indicating individual differences in liver response to this toxicant. We further quantified the relationship between observed changes in gene expression between toxicants by calculating the Pearson’s correlation coefficient (r) for each pair of toxicants (Figure 2b,c). Consistent with the clustering analysis, the analysis captured the correlation between APAP and BB both at the short time point (5 h, r = 0.45) and at the later time point (10 h, r = 0.49) when we used all common genes between each of the two toxicants (Figure 2b). Our results also showed a strong correlation between CCl_4_ at the early time point with respect to changes observed for APAP at the later time point, indicating similarities in liver responses despite differences in the time after exposure. However, when we used only significantly altered genes that are common between each time point (false discovery rate (FDR) < 0.1), we found an improved correlation between the changes observed for APAP and BB at both time points (Figure 2c). Interestingly, in this case, we also found an improved correlation between changes for CCl_4_ at the short time point with that of the later time point for both APAP (r = 0.80) and BB (r = 0.58). These results indicate that CCl_4_-induced peak alterations in the liver metabolism occurred earlier than the other two toxicants. We provide a complete list of all genes in each study together with their significance values in Supplementary Table S1.

**Figure 3:**
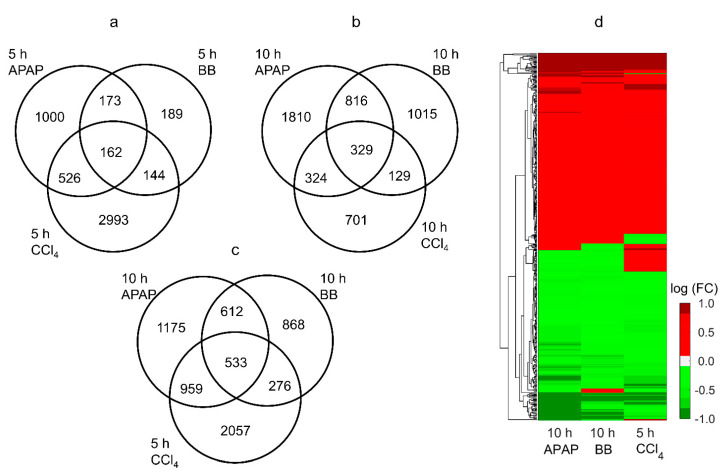
Venn diagrams depicting the commonalities among the significantly changing gene expressions for all three toxicants (APAP, BB, and CCl_4_) at (**a**) the 5 h time point, (**b**) the 10 h time point, and (**c**) based on the time point at which peak gene expression alterations were observed for each toxicant. (**d**) Heat map of the significantly (FDR < 0.1) altered common genes in the liver based on the peak alteration time points for each toxicant. Red and green bars indicate up- and down-regulated genes, respectively.

Among the total genes detected for each toxicant and exposure time combination, we identified the highest number of significantly (FDR < 0.1) changed genes (3825) for CCl_4_ at the earlier time point. Figure 3 shows a summary of genes that changed significantly for each chemical and exposure time point and the number of genes that were common between them. In contrast to the CCl_4_ study, APAP and BB induced the largest number of significantly changed genes (3279 and 2289, respectively) at the later time point (10 h). We identified 162 and 329 genes that were common for all toxicants at the short and later time points, respectively (Figure 3a,b). Interestingly, when comparing alterations at the peak response for each toxicant, we identified the largest number of significantly changing genes (533) common between them (Figure 3c). A heat map of logarithmic FC values of these common genes at the peak response indicated an average pairwise correlation of 0.84 (standard deviation (SD) = 0.07) between the changes across these three toxicants (Figure 3d). We observed commonalties in several up- and down-regulated genes that changed similarly across the toxicants, indicating their crucial role in inducing liver toxicity. We provide all the common genes across these toxicants under the studied conditions in Supplementary Table S2.

### 2.2. Toxicant-Induced Changes in Gene Expression Predict Liver Injury Phenotypes

**Figure 4:**
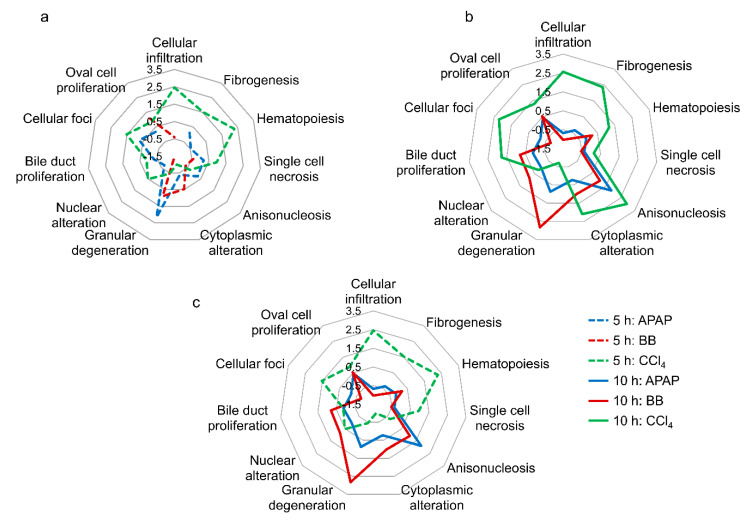
Radial diagrams indicating liver injury phenotypes (histopathology) based on significantly altered genes identified for each toxicant (FDR < 0.1). Liver injury module z-score values at (**a**) the 5 h time point (dashed lines), (**b**) at the 10 h time point (solid lines), and (**c**) based on the time points at which peak alterations are observed in liver gene expression.

Using publicly available chemical exposure data, we previously developed 11 liver injury modules based on co-expressed gene sets associated with specific histopathological injury phenotypes in the liver [27,28]. To test the predictive capability of the significantly altered genes in activating a specific liver injury module, we calculated the modules z-score values based on an absolute aggregated fold change (AAFC) method (see Method Section for details). The injury modules with largest z-score were then considered as the most probable histopathology phenotype for a specific toxicant and sample time point. Figure 4 shows a radial graph of activation scores for liver injury modules for each toxicant and time point. The analysis indicated that, at the early time point, CCl_4_ exposure maximally activated injury modules associated with cellular infiltration, hematopoiesis, and cellular foci, while APAP and BB activated granular degeneration (Figure 4a). We observed increases in module activation scores at the later time point, further implicating fibrogenesis and anisonucleosis as injury phenotypes for CCl_4_ and anisonucleosis for APAP and BB exposure (Figure 4b,c). These results show that exposure to CCl_4_ leads to activation of injury-specific pathways in cellular inflammation (cellular infiltration, fibrogenesis, and cellular foci) and cellular proliferation (cellular foci), which constitute the initiating factors for liver fibrosis [30,31]. Similarly, APAP and BB exposure leads to activation of cellular degeneration (granular degeneration and anisonuleosis), indicative of a necrotic end-point [32,33,34].

### 2.3. Correlation between Plasma Metabolite Profiles

**Figure 5:**
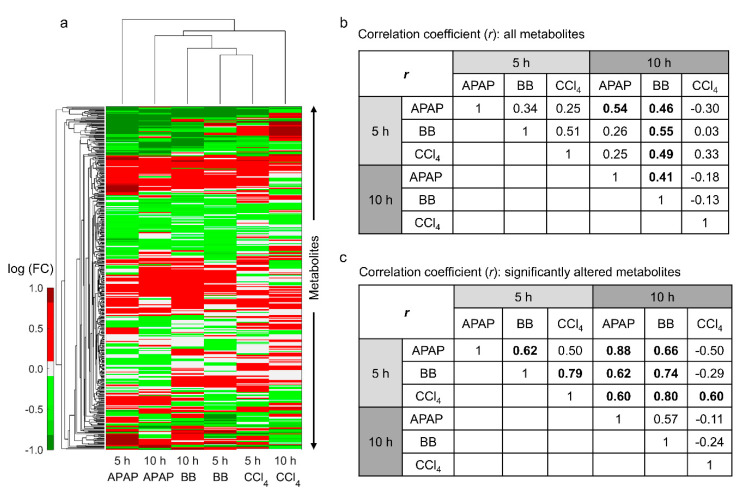
Identification of similarities in plasma metabolite levels from rats exposed to either APAP, BB, or CCl_4_. (**a**) Hierarchical clustering analysis of all common metabolites in plasma at 5 and 10 h after exposure of the toxicant. Pairwise correlation between the natural logarithmic fold changes (log(FC)) in metabolites observed across all three toxicants (**b**) without using any significance cut-off values and (**c**) with using only significantly altered metabolite levels (FDR < 0.1). Pairwise combinations with high correlation coefficient values are indicated in bold.

Analogous to the gene expression study, we looked at the commonalities in plasma metabolite profiles across the toxicants and exposure time points. We identified the maximum perturbations in the BB study, with 446 and 428 (out of 735) metabolites significantly changed at 5 and 10 h post exposure (FDR < 0.1), respectively [25]. Similarly, for APAP, we identified 220 and 165 out of 569 [22] respectively, and for CCl_4_, we identified 170 and 136 out of 645 metabolites, respectively (Supplementary Table S3). Of the total number of metabolites detected in each study, we identified 449 metabolites that were common for the three toxicants. Figure 5 shows a hierarchical clustering analysis of logarithmic FC values of all common metabolites for each toxicant exposure relative to their controls. Unlike changes in gene expression profiles, changes in plasma metabolites were closely clustered together for the two sampled time points for the same chemical (Figure 5a,b). However, we did observe clustering between BB and CCl_4_ at the short time exposure (5 h), indicating greater similarities in plasma metabolite changes between them. Indeed, the calculated pairwise correlation coefficient for BB and CCl_4_ at 5 h was 0.51 (Figure 5b).

**Figure 6:**
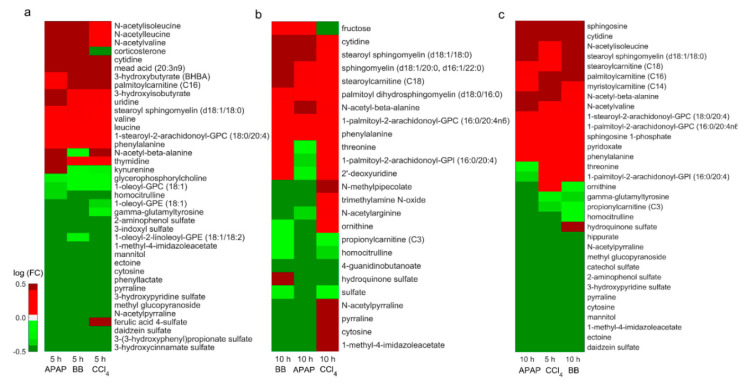
A heat map of significantly altered plasma metabolites (FDR < 0.1) that were common to APAP, BB, and CCl_4_ exposures (**a**) at the 5 h time point, (**b**) at the 10 h time point, and (**c**) based on time points at which we observed maximum correlation. Red and green bars indicate elevated and decreased metabolite levels, respectively.

We further evaluated similarities between these toxicant exposures by comparing significantly (FDR < 0.1) altered metabolites (Figure 5c). These results show that the highest similarity between different toxicants occurs for CCl_4_ at the early time point and BB (r = 0.80) and APAP (r = 0.60) at the later time point. These shared metabolite changes suggest that we can identify similarities in liver response to different toxicants, despite differences in their exposure times. Figure 6 shows a heat map of the common metabolites that changed significantly across the three toxicants. In particular, we were able to identify 39 and 25 metabolites common to all toxicants at the earlier time point (Figure 6a) and at the later time point (Figure 6b), respectively. We observed a conserved metabolite signature at the earlier time point across the three toxicants with increases in several amino acid- and lipid-related metabolites and decreases in several sulfated metabolites. However, we did not see a consistent signature at the later time point, with changes similar between APAP and BB but differing for CCl_4_, reflecting differences in the underlying toxicity mechanisms. However, we observed correlations between metabolite changes for CCl_4_ at the early time point with those occurring at the later time point for APAP and BB (Figure 6c). Thus, the highest similarities observed in this study occurred when the toxicants induced the maximum perturbations.

### 2.4. Toxicants Differed in Altering Hepatic Fluxes in Central Carbon Metabolism

**Figure 7:**
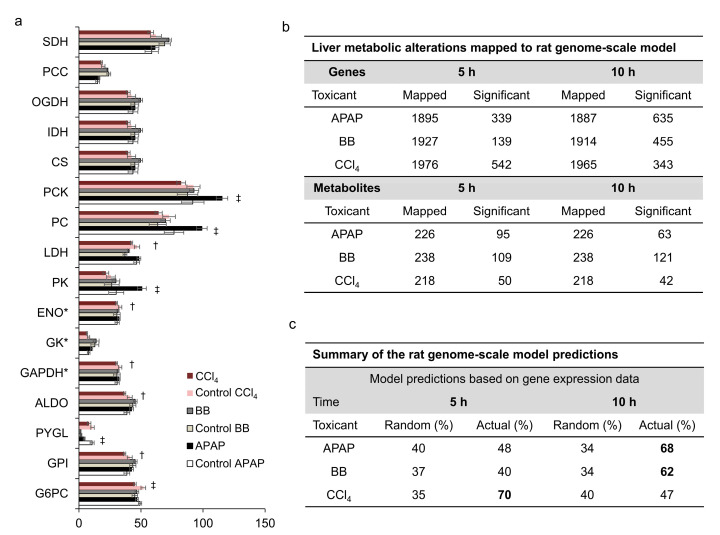
Absolute hepatic flux changes induced by either APAP, BB, or CCl_4_, and metabolic network predictions of metabolite-level changes based on the measured liver gene expression values. (**a**) Bar graphs of flux measurements calculated from metabolic flux analysis 10 h after treatment with either APAP (black), BB (dark grey), or CCl_4_ (maroon) compared to their vehicle groups (white, light grey, or pink, respectively). Abbreviations: ALDO, aldolase; CS, citrate synthase; ENO, enolase; GAPDH, glyceraldehyde-3-phosphate dehydrogenase; GK, glycerol kinase; GPI, D-glucose-6-phosphate isomerase; G6PC, D-glucose-6-phosphatase; IDH, citrate dehydrogenase; LDH, lactate dehydrogenase; OGDH, oxoglutarate dehydrogenase; PC, pyruvate carboxylase; PCC, propionyl-CoA carboxylase; PCK, phosphoenolpyruvate carboxykinase; PK, pyruvate kinase; PYGL, glycogen phosphorylase; SDH, succinate dehydrogenase (^‡^ *p* < 0.05, ^†^ 0.05 < *p* < 0.1, and * value in Hexose units). (**b**) Summary of the total and significantly altered liver genes (FDR < 0.1) and plasma metabolites mapped to the rat metabolic network model. (**c**) Summary of predictions for changes in plasma metabolite levels based on changes in liver gene expression.

Although we observed correlations between different toxicants when assessing changes in liver gene expression and plasma metabolites, we did not see similar levels of concordance in the measured liver metabolic fluxes. Figure 7a shows that in the APAP study, liver pyruvate cycling was elevated and glycogenolysis was reduced (*p* < 0.05) at the later time point. In contrast, we observed a significant decrease in several glucose-producing fluxes downstream of enolase in the CCl_4_ study, but did not observe significant differences in the BB study. These results indicate that each toxicant altered hepatic glucose-producing fluxes differently based on their different mechanisms of toxicity. Furthermore, our flux measurements indicated only minor contributions from glycogen (PYGL in Figure 7a) compared to that of gluconeogenesis pathways at 10 h post-toxicant exposure under fasting conditions. Majority of these fluxes in glucose production are in the periportal area of hepatic lobules where the effect of toxicants is minimum. Therefore, we speculate that this could be one of the reasons we did not see major perturbations in hepatic fluxes in the glucose production pathway.

### 2.5. Identification of Metabolic Changes in Liver Metabolism Associated with Liver Toxicity

Our global analysis identified changes in liver metabolism that were specific to and common across different toxicant exposures. However, these changes in genes and metabolites were highly interlinked, and the analysis did not provide mechanistic explanations for the observed changes. Therefore, we used the rat genome-scale metabolic network model to first identify metabolic genes that drive major enzymatic reactions in liver metabolism and subsequently connected those genes to associated plasma metabolites. Figure 7b shows a summary of metabolic genes and metabolites that were represented in the metabolic model for each toxicant and exposure time points. Thus, for the CCl_4_ exposure, we mapped the maximum number of significantly changed genes (542) to the earlier time point; similarly, for the APAP exposure, we mapped a maximum number of genes (635) to the later time point. The number of genes mapped to the model were lowest for BB study at both time points when compared with the other two toxicants. In contrast, when we compared the changes in metabolites mapped to the model for the three toxicants, we identified the maximum number of significantly altered plasma metabolites in the BB study at both time points. These results suggest that although gene-expression changes were one of the major drivers for changes in metabolites, there exists a non-linear relationship between liver genes and plasma metabolites with multiple factors contributing to the observed changes.

To identify contributions of liver gene-expression changes that drive major changes in plasma metabolites, we used genes mapped to the metabolic network model in each study and applied the transcriptionally inferred metabolic biomarker response (TIMBR) algorithm [18] to predict the capability of the metabolic network to either produce or consume each metabolite. To ensure that the network realistically captures metabolite changes, we imposed experimentally observed changes in absolute flux values from the MFA analysis as constraints on the model. A comparison of model predictions with experimental observations indicated that, of the total number of metabolites that significantly changed and could be mapped to the network model, we identified approximately 65% of them as causally linked to the liver gene-expression changes for APAP and BB at the later time point and 70% for CCl_4_ at the earlier time point (Figure 7c). In contrast, when we provided the network with random gene-expression changes as inputs, the direction of change was only correctly predicted for 34% of the same metabolites, indicating that gene-expression changes are a major contributing factor in driving changes in specific metabolite levels. Furthermore, our results suggest that the causal linkage for the observed metabolite changes was better captured by the model when significant changes in liver gene expression occurred compared with moderate changes. Low levels of gene expression changes resulted in a lower correspondence between the model predictions and experimental observations. Here, the model predicted only approximately 45% of the total metabolites correctly compared to an average random prediction of 39% for the three toxicants (Figure 7c).

### 2.6. Common Changes in Amino Acid and Lipid Metabolism as Potential Signatures of Liver Injury

To identify common metabolic signatures of liver injury in response to toxicant exposure, we further used the genes mapped to the metabolic network in each study and identified metabolic pathways that are specific to each study and common across them. In particular, we selected significantly changed genes for each toxicant at each exposure time point and performed a pathway level analysis (see Methods for details). Table 1 shows the summary of the highly enriched pathways for the toxicant exposure conditions at which we observed peak gene perturbations in liver metabolism together with their directionality (up- or down-regulated) relative to control studies. We arranged the metabolic pathways based on the number of different toxicants that produced significant changes in gene expression (all three, or just two out of three). We observed similarities for lipid metabolism with several genes in its subordinate pathways being significantly altered across the three toxicants. For example, we identified glycerolipid and glycerophospholipid metabolism as involving the largest number of altered genes, and the associated aggregate fold change (AFC) values indicated that a majority of the genes in these pathways were upregulated. Similarly, we identified significantly altered pathways in steroid and fatty acid metabolism, but with the majority of genes in these pathways being downregulated, indicating a common dysregulation in lipid metabolism due to toxicant exposure. We observed similar behavior with respect to changes in amino acid metabolism, where individual subordinate pathways were consistently upregulated across the three toxicants, except for tryptophan metabolism. However, we did not observe a consistent signature for changes in either carbohydrate or nucleotide metabolism, indicating toxicant-specific alterations among these pathways.

Using the metabolite predictions of the metabolic network model together with pathway level analysis, we compiled a panel of significantly altered plasma metabolites that were correlated with changes in genes’ expression. Table 2 shows the list of these metabolites and their similarities across the three toxicants based on time points at which we observed the maximum correlation between them (Figure 5c). In particular, based on the consistent gene-expression changes among lipid metabolism-related pathways, our analysis identified several metabolites, such as palmitoyl and stearoylcarnitine, stearoyl sphingomyelin, sphingosine, and sphingosine-1-phosphate, which were consistently increased in the plasma. In contrast, in the amino acid-related pathways, we observed methylimidazoleacetic acid as the only common metabolite that was significantly decreased among the metabolites that mapped to the model, despite the observation of greater similarities in gene expression at the tissue level. Furthermore, a positive TIMBR score value for this metabolite in the BB and CCl_4_ studies indicated that the decrease might not be directly associated with changes in gene expression. Similarly, we identified several metabolites in amino acid, nucleotide, and co-factor metabolism pathways that were consistent in at least two toxicant exposures and predicted correctly by the model using TIMBR scores. These results suggest that the metabolic pathways (and their metabolites) that are affected by at least two toxicants could be mined for potential common indicators of liver toxicity.

## 3. Discussion

To gain insights into the underlying molecular mechanisms of liver toxicity, we probed the early response in liver metabolism arising from acute toxicity induced by three model liver toxicants in rats. We selected toxicant dose levels that induced liver toxicity within 36 h post administration of a single dose. To capture and probe the initial responses, we selected two early time points (5 and 10 h) at which traditional liver functional markers were not yet elevated (Supplementary Figure S1). The similar experimental conditions used and data types collected for all three toxicants gave us the ability to analyze concurrently measured changes in genes and metabolites induced by each toxicant and provide a consistent comparison between them. Furthermore, the use of genome-scale metabolic network modeling enabled us to interpret systemic effects, provide deeper insights into the multi-omics data, understand the genotype-to-phenotype relationships for injury-specific pathways, and identify the associated metabolites. Based on these analyses, we proposed several genes and plasma metabolites linked to lipid and amino acid metabolism and common to the different toxicant exposures as potential mechanism-based indicators of liver toxicity.

Our analysis showed that changes in gene expression could be highly correlated between the toxicants (Figure 2c), indicative of major similarities in the mechanism of toxicity. In particular, we observed stronger correlation between APAP and BB compared to that of CCl_4_. Interestingly, we observed similar correlations between APAP and BB with respect to CCl_4_ when comparing CCl_4_ changes at the earlier time with those for the later time points of APAP and BB, indicating that the peak in CCl_4_-induced liver toxicity occurred earlier. Despite differences in the experimental conditions, previous studies have shown a similar behavior for CCl_4_ toxicity as the observed peak perturbations occur within 6 h of a single dose of CCl_4_ [24]. Furthermore, we identified similar trends for changes in plasma metabolite levels, with a maximum correlation observed at the 5 h time point for CCl_4_ with that of the 10 h time point for APAP and BB (Figure 5c). Based on comparisons at the observed peak response times, we identified a common set of 533 genes that changed similarly across all three toxicants and that might play a role in subsequent liver injury (Figure 3d and Supplementary Table S2). We list here 5 upregulated (Hmox1, Trim80, Maff, Adm2, and Trib3) and 5 downregulated genes (Aox3, Nfe2, Stac3, Nrep, and Asap3) that are common between the toxicants at the peak response out of the top 50 common genes based on their fold change values for each toxicant. Products of some of these genes have previously been reported as potential indicators of liver toxicity [35]. Similarly, we identified a common set of altered plasma metabolites that were mechanistically linked via the metabolic network to a phenotypic liver response (histopathology). These changes were also observed among the circulating metabolites in plasma and, hence, can be proposed as potential indicators of liver toxicity (Figure 6).

Our analysis of changes in hepatic flux in the glucose production pathway captured potential differences between the toxicants’ mechanism of action. For example, our analysis showed a major difference in the pyruvate cycling for APAP exposure but not for CCl_4_ and BB. These findings are consistent with the differences that we observed at the gene level analysis in the carbohydrate metabolism for these toxicants (Table 1). For example, changes in genes in glycolysis/gluconeogenesis and pyruvate metabolism pathways were differently regulated across the three toxicants, indicating differences in the effect of these toxicants on the glucose production. Similarly, we did not see many similarities in the plasma metabolites’ changes in carbohydrate metabolism (Table 2) across these toxicants, which also emphasizes major differences in the glucose production pathway based on their mechanism of toxicity. Furthermore, the low concordance observed in hepatic fluxes across these toxicants compared to changes in genes and metabolites could be attributed to several factors. First, the richness and size of the transcriptomics and metabolomics allows coverage of many pathways that were not monitored at the flux level. Second, expression changes alone may not lead to changes in hepatic fluxes since metabolic flux is controlled by additional mechanisms, such as allosteric feedback, post-translational modifications, and substrate/cofactor availability [36].

Using our previously developed liver injury modules [27,28], we were able to evaluate the capability of gene expression changes in the liver to predict toxicant-specific injury phenotypes, such as necrosis and fibrosis, in the case of CCl_4_ (Figure 4). For example, in the case of an acute overdose, APAP and BB are known to cause necrotic liver toxicity due to a shortage of glutathione (GSH) required to detoxify the toxic metabolites N-acetyl-p-benzoquinone imine and 3,4-epoxide, respectively [37,38]. Similarly, at high doses, CCl_4_ is also a potent hepatotoxicant leading to either necrosis or steatosis, as well as fibrosis when breakdown metabolites, such as trichloromethyl free radicals, covalently bind to cell proteins [39]. Our analysis provided a new and efficient strategy to identify early and commonly occurring perturbations in liver metabolism using known model liver toxicants. Other uncharacterized chemicals or drugs exhibiting similar changes could then be hypothesized to be associated with acute liver toxicity if given in excessive doses.

Although our clustering-based analysis showed commonalties among genes and metabolites across the toxicants, it cannot provide a direct mechanistic linkage between them. However, the use of a genome-scale metabolic network model that links changes in gene expression and metabolite levels provides a framework to assess systemic mechanisms of liver toxicity. For example, we were able to identify several pathways in lipid and amino acid metabolism that changed similarly across all three toxicants and were associated with lipid peroxidation and GSH depletion (Table 1). Thus, we linked downregulated genes in fatty acid and steroid metabolism to dysregulation in mitochondria and increased lipid peroxidation. Similarly, to overcome GSH depletion, genes in amino acid metabolism were upregulated to support re-synthesis of GSH using precursor molecules from this pathway. Furthermore, our metabolic network modeling analysis allowed us to link apparent changes in plasma metabolite levels to changes in gene expression. Out of all metabolites that we could map to the metabolic network, we correctly predicted the directional change for more than 60% of the metabolites detected in the plasma (Figure 7c). This provides support for a mechanistic link between changes in gene expression and circulating plasma metabolites. That is, metabolites associated with dysregulation due to lipid peroxidation and consistently increased in plasma for all toxicants (Table 2) could be monitored to gauge liver health.

Indeed, several previous studies have indicated alterations in lipid metabolism, such as alteration in plasma carnitine levels and sphingolipid metabolites, with chronic liver disease and liver cirrhosis patients [40,41,42,43,44]. The acute exposure of the studied chemicals led to a predominant downregulation of several genes involved in fatty acid metabolism (Table 1), indicative of mitochondrial dysfunction. Consistent with impairment of mitochondrial β-oxidation, we found acylcarnitines, such as palmitoylcarnitine and stearoylcarnitine, consistently exhibiting increased plasma levels across the three toxicants (Table 2). Acylcarnitines play an important role in liver cellular metabolism and are involved in transport of long-chain fatty acids into mitochondria for β-oxidation to provide energy for cellular activities [45]. Similarly and consistent with altered sphingolipid metabolism, our analysis showed a consistent increase in sphingosine-related metabolites, such as sphingosine and sphingosine-1-phosphate. Because the liver is an important regulator of sphingosine levels in the blood, alterations in sphingosine-1-phosphate levels are known to be associated with fatty liver disease [46,47] and liver fibrosis [48]. Hence, monitoring early perturbations in plasma concentration of these common metabolites may provide early indications of the initiation and onset of liver toxicity.

In summary, using a systems biology approach, we probed alterations in rat liver metabolism common to different hepatotoxicants and identified global changes in liver gene expression as well as plasma metabolite levels. We identified significant commonalities between gene-expression changes in the liver induced by the three toxicants, and the observed correlations between them were maximal when probed at their peak response. We showed that the identification of early gene perturbations post-toxicant exposure could be used to predict impending liver injuries. Furthermore, using an integrated metabolic network modelling approach, we identified several common injury-specific pathways and metabolites within them whose alterations were causally related to known mechanisms of toxicity. In particular, we identified several toxicant-specific and common metabolites involved in lipid and amino acid metabolism, which could be monitored as potential indicators of toxicant-induced liver damage. Therefore, our results demonstrated that the metabolic network-based systems biology approach can serve as a tool to integrate high-throughput data from multiple toxicants, elucidate the underlying mechanisms of chemical-induced toxicity, and suggest strategies to identify new indicators of liver injury.

## 4. Materials and Methods

### 4.1. Animals and Toxicant Dose Determination

We carried out all the experiments in accordance with the Guide for the Care and Use of Laboratory Animals of the United States (U.S.) Department of Agriculture and the National Institutes of Health, after obtaining protocol approval from the Vanderbilt University Institutional Animal Care and Use Committee (M1600175-00, 08/26/2016) and the U.S. Army Medical Research and Development Command Animal Care and Use Review Office (13012001, 11/21/2016). We used male Sprague Dawley rats at 10 weeks of age (approximately 280–320 g) purchased from Charles River Laboratories (Wilmington, MA, USA) and housed them under environmentally controlled conditions (12 h light:12 h dark cycle at 23 °C). We allowed the rats free access to water and a commercially available rodent diet, Formulab Diet 5001 (LabDiet, Richmond, IN, USA), and all animals were acclimated to these housing conditions minimally for a week prior to any experiments. Seven days before each experiment, we anesthetized rats with isoflurane and performed a catheter implantation surgery for sample collection. To determine the appropriate dose and exposure time for assessment after each toxicant, we used oral gavage to administer either vehicle or toxicant at different dose levels and collected blood samples at regular intervals. We then measured the plasma levels of ALT and AST to evaluate acute liver toxicity and selected an optimal effective dose and exposure time to assess systematic perturbations in liver metabolism. We refer the reader to details of this procedure in our recent publications [22,25,26].

### 4.2. Studies for Measuring Changes in Liver Gene-Expression and Plasma Metabolic Profiles

Based on the results of dose determination studies, we selected 2.0, 0.4, and 0.3 g/kg as the appropriate doses for APAP, BB, and CCl_4_, respectively. We selected two assessment times after exposure, one early (5 h, n = 8) and one later (10 h, n = 8), to obtain transcriptomic and metabolomic data. For each study, we gave animals either vehicle (6 mL/kg of 50% polyethylene glycol, 5 mL/kg of corn oil, and 2 mL/kg of corn oil for APAP, BB, and CCl_4_, respectively) or one of the toxicants at the selected dose and collected blood and liver tissue samples for metabolic profiling and RNA-sequencing. We anesthetized rats by intravenous injection of sodium pentobarbital through a jugular vein catheter, removed the liver and froze it using Wollenberger tongs precooled in liquid nitrogen. We stored the collected plasma and liver samples at −80 °C until used for analyses.

For RNA-sequencing, we isolated total RNA from the liver using TRIzol Reagent (Thermo Fisher Scientific, Waltham, MA, USA) and the direct-zol RNA MiniPrep kit (Zymo Research, Irvine, CA, USA). We subjected the pooled libraries to 75 bp single-end sequencing for the APAP study (HiSeq3000; Illumina, San Diego, CA, USA) and 150 bp paired-end sequencing for the BB and CCl_4_ study (NovaSeq6000; Illumina, San Diego, CA, USA) according to the manufacturer’s protocol. For metabolomics studies, we subjected individual plasma samples to methanol extraction and divided them into aliquots for analysis using ultra-high-performance liquid chromatography/MS (UHPLC/MS). We refer the reader to our previous publications for further details [25,26].

### 4.3. Analysis of RNA-Sequencing and Metabolomic Data

We used the RNA-sequencing data analysis pipeline Kallisto/Sleuth for read alignment and quantification, and differential analysis [49,50]. Kallisto pseudo-aligns the reads to a reference, producing a list of transcripts that are compatible with each read while avoiding alignment of individual bases. Kallisto employs a bootstrapping technique to calculate uncertainties of transcript abundance estimates by repeating (100 times) the analysis after resampling with replacement. In this study, we pseudo-aligned the reads to the latest version of *Rattus norvegicus* transcriptome (Rnor_6.0) downloaded from the Ensembl website [51]. We analyzed the Kallisto-derived transcript abundance files for differential gene expression using Sleuth. Briefly, we first prepared an auxiliary table that describes the relationship between the Kallisto-derived abundance files to control and treatment samples and constructed a Sleuth object, which stores not only information about the experiment, but also details of the (full) model to be used for differential testing. We then performed a differential analysis using the Wald test to obtain the estimated logarithmic fold changes between treatment and control samples.

We performed two types of statistical analyses for metabolite changes: (1) significance tests and (2) classification analyses. For all statistical analyses, which were based on log-transformed data, we used ArrayStudio and customized programs in R (http://cran.r-project.org). Following log transformation and imputation of missing values (if any) with the minimum observed value for each compound, we used Welch’s 2-sample t-test to identify metabolites that differed significantly (*p* < 0.05) between experimental groups. We estimated the false discovery rate (FDR: q value) to correct for multiple comparisons.

### 4.4. Tracer Labeling Studies for Measuring Metabolic Flux and Metabolic Flux Analysis

For each study, at 7 a.m. on the day of the study, we administered either vehicle or toxicant (n = 8 each) to the rats as described above for transcriptomic studies. At 12:50 p.m., we anesthetized the animals with isoflurane and, during this short period of anesthesia (approximately 5 min), collected 200 μL of arterial blood through the carotid artery catheter to determine the natural isotopic abundance of circulating glucose. We then subcutaneously administered a bolus of [^2^H_2_] water (99.9%) containing 0.9% sodium chloride to enrich total body water to 4.5%. After they had recovered from anesthesia, we placed the rats in bedded containers without food and water, and then connected them to sampling and infusion lines. At 1 p.m. (i.e., 6 h after toxicant administration), we delivered [6,6-^2^H_2_]glucose (80 mg/kg prime + 0.8 mg/kg/min infusion) as a prime-constant infusion into the systemic circulation through the jugular vein catheter for the duration of the study. Starting 120 min after the [^2^H_2_] water bolus, we delivered sodium [^13^C_3_] propionate (99%) as a prime-constant infusion (110 mg/kg + 5.5 mg/kg/min infusion). We prepared all infusates in a 4.5% [^2^H_2_] water-saline solution unless otherwise specified. Stable isotopes were obtained from Cambridge Isotope Laboratories (Tewksbury, MA, USA). At each of the three time points during the last 20 min of the tracer-infusion period (100, 110, and 120 min from the start of the infusion), we collected a 300 μL arterial blood sample in an EDTA-coated tube. We centrifuged the collected blood samples immediately to isolate plasma samples, which we stored at −80 °C for further analyses. We performed glucose derivatization and gas chromatography-mass spectrometry (GC–MS) analysis as described previously [25,26].

To determine the absolute hepatic fluxes in the glucose production pathway, we employed the in vivo MFA methodology described previously [25,26,52]. Briefly, we constructed a reaction network using the INCA software package [53] and defined the carbon and hydrogen transitions for biochemical reactions linking hepatic glucose production and its associated intermediary metabolic reactions. INCA relies on the elementary metabolite unit method to simulate mass isotopomer distributions of measured metabolites and to regress the metabolic network model to fit the experimental measurements. We estimated the flux through each reaction relative to citrate synthase (fixed at 100) by minimizing the sum of squared residuals between the simulated and experimentally determined mass isotopomer distributions of six glucose fragment ions, and repeated this process 50 times by randomizing the initial values. We converted the relative fluxes to absolute values using the known [6,6-^2^H_2_] glucose infusion rate and rat weights, and then averaged the flux estimates for the steady-state samples to obtain a representative set of values for each rat.

### 4.5. Rat Metabolic Network and Algorithm for Data Integration and Metabolite Predictions

We used the *R. norvegicus* genome-scale metabolic network model [29] and a previously developed algorithm, transcriptionally inferred metabolic biomarker response (TIMBR), for high-throughput data integration [18] to predict plasma metabolite changes based on changes in liver gene expression. Briefly, the TIMBR algorithm uses the gene-protein-reaction relationships in the model to convert the log_2_ fold changes of liver gene expression into reaction weights. It then calculates the global network demand required for producing a metabolite in the blood. The objective function minimizes the weighted sum of fluxes across all reactions for each condition and metabolite, to satisfy the associated mass balance and the optimal fraction of the maximum network capability to produce a metabolite. We then imposed the measured MFA values as constraints on the model as upper and lower bounds for the respective reactions in the glucose production pathway. Based on values reported in the literature, we used appropriate uptake and secretion rates for the exchange reactions of the liver and kidney under short-term fasting conditions [26]. Thus, using the gene-expression changes together with the uptake and secretion rates along with MFA constraints, TIMBR provides a production score (z-score) that represents an increase or decrease for each metabolite in the plasma and urine.

We used the experimental log_2_ fold changes of significantly altered (FDR < 0.1) plasma metabolites from the global metabolic profiling data and then compared the corresponding TIMBR production scores from the rat genome-scale model at 5 or 10 h post-toxicant treatment. Here, we considered the metabolite levels as having increased or decreased based on TIMBR production score cut-off values of greater than 0.1 and smaller than –0.1, respectively. We considered metabolites with scores between –0.1 and 0.1 as unchanged. To test the robustness of the results from the multi-tissue model, we randomized the original gene-expression data by randomly sorting the gene names and using the resulting data as the input.

### 4.6. KEGG Pathway Analysis

To understand the biological significance of changes in gene expression levels, we calculated the significantly activated KEGG pathways by using the online tool Database for Annotation, Visualization, and Integrated Discovery (DAVID) [54] and a list of significantly altered metabolic genes for each toxicant as the input. In addition, we used the aggregated fold change (AFC) method [55], which calculates significantly enriched KEGG pathways together with their direction of change, to ascertain that the results were independent of the pathway-detection method. Briefly, the AFC method calculates the mean fold change (FC) value for each gene and defines the KEGG pathway score as the total FC value of all genes in the pathway. The sign of the pathway score represents the direction of regulation, with positive values indicating upregulation and negative values indicating downregulation in the treatment condition compared to their corresponding controls.

### 4.7. Liver Injury Module Activation Score

We used the aggregated absolute FC (AAFC) method to calculate the activation score of liver injury (histopathology) modules that are significantly changed [27,56]. Briefly, the AAFC method first calculates the absolute value of each gene’s log-transformed FC value, and then the total FC value of the absolute values for each module. We then used the gene set scores to perform null hypothesis tests and estimated each gene set’s significance by its *p*-value, defined as the probability that the score for randomly selected FC values (10,000 times) is greater than the score from the actual gene set. A small *p*-value implies that the gene set value is significant. The z-score is the number of standard deviations by which the actual gene set value differs from the mean of the randomly selected FC values (10,000 times). In this study, we used only significantly (FDR < 0.1) changed genes for each toxicant to determine the module activation scores.

## Figures and Tables

**Table 1:** Summary of the significantly altered common metabolic pathways identified using gene-enrichment analysis at the peak response. Values in bold represent pathways that were consistently up- or down-regulated across all three toxicants. Positive and negative aggregated fold change (AFC) z-scores indicate pathways that are up- and down-regulated, respectively. AA: Amino acid; Carb: Carbohydrate.

Main Pathway	Subordinate Pathway	10 h: APAP		10 h: BB		5 h: CCl_4_	
		Genes	AFC z-score	Genes	AFC z-score	Genes	AFC z-score
**Lipid**	**Glycerophospholipid metabolism**	**28**	**1.42**	**26**	**1.75**	**21**	**0.10**
	Glycerolipid metabolism	18	0.31	15	2.84	14	−0.97
	**Steroid biosynthesis**	**12**	**−4.42**	**13**	**−4.09**	**6**	**−1.58**
	**Steroid hormone biosynthesis**	**26**	**−3.49**	**11**	**−1.01**	**11**	**−0.90**
	**Fatty acid metabolism**	**14**	**−1.89**	**17**	**−1.83**	**12**	**−1.96**
	**Biosynthesis of unsaturated fatty acids**	**10**	**−1.23**	**13**	**−0.21**	**7**	**−1.63**
	**Fatty acid degradation**	**14**	**−0.03**	**12**	**−0.07**	**8**	**−2.06**
	Arachidonic acid metabolism	15	−0.42	12	−0.71		
	Fatty acid elongation	8	1.08	9	1.30		
	Sphingolipid metabolism	12	0.96			13	−1.81
	Ether lipid metabolism			7	−0.96	12	0.03
	Fatty acid biosynthesis			5	−1.57	5	−0.93
	Primary bile acid biosynthesis			5	0.57	5	−1.49
**AA**	**Glycine, serine, and threonine metabolism**	**17**	**1.63**	**7**	**1.15**	**14**	**1.51**
	**Cysteine and methionine metabolism**	**17**	**2.16**	**13**	**1.47**	**7**	**1.26**
	**Arginine and proline metabolism**	**12**	**0.45**	**12**	**0.40**	**12**	**1.30**
	**Glutathione metabolism**	**17**	**0.42**	**8**	**0.76**	**13**	**2.94**
	**Alanine, aspartate, and glutamate metabolism**	**11**	**2.28**	**7**	**2.15**	**11**	**0.55**
	**Tryptophan metabolism**	**15**	**−3.63**	**10**	**−1.46**	**11**	**−1.99**
	Arginine biosynthesis	6	2.40			5	0.06
	Selenocompound metabolism	8	−1.03			5	1.29
	Valine, leucine, and isoleucine degradation			10	−2.33	16	−2.16
	beta-Alanine metabolism			6	0.71	11	0.75
**Carb**	Amino sugar and nucleotide sugar metabolism	15	−1.40	15	−2.64	12	2.46
	Phosphatidylinositol signaling system	14	−0.10	13	1.54	19	−1.73
	**Fructose and mannose metabolism**	**10**	**0.31**	**12**	**0.19**	**8**	**2.85**
	**Starch and sucrose metabolism**	**9**	**−1.97**	**8**	**−2.03**	**9**	**−0.44**
	**Glyoxylate and dicarboxylate metabolism**	**11**	**−1.56**	**11**	**−2.21**	**10**	**−1.44**
	Glycolysis/Gluconeogenesis	18	0.18	18	−2.22	13	1.17
	Pyruvate metabolism	17	−0.67	15	−2.36	7	0.13
	Ascorbate and aldarate metabolism	8	−1.12	6	−0.40		
	Propanoate metabolism			7	−1.52	9	−1.39
	Pentose phosphate pathway			10	−0.41	7	1.46
**Nucleotide**	**Purine metabolism**	**44**	**0.39**	**24**	**0.52**	**43**	**0.09**
	Pyrimidine metabolism	29	−0.57	15	−0.15	24	0.08

**Table 2:** Summary of the model predictions for significantly altered plasma metabolites in the injury-specific pathways that are common for at least two toxicants. Metabolite entries in bold indicate a consistent direction of fold change among all three toxicants.

		10 h: APAP		10 h: BB		5 h: CCl_4_	
Pathway	Metabolite	log(FC)	TIMBR Score	log(FC)	TIMBR Score	log(FC)	TIMBR Score
**Lipid**	**L-palmitoylcarnitine**	**0.52**	**0.18**	**0.68**	**0.58**	**0.70**	**0.70**
	**stearoyl sphingomyelin (d18:1/18:0)**	**0.83**	**0.75**	**0.78**	**0.69**	**0.39**	**0.07**
	**sphingosine**	**0.62**	**1.81**	**1.36**	**0.43**	**1.20**	**0.15**
	**sphingosine-1-phosphate**	**0.37**	**1.73**	**0.41**	**0.43**	**0.32**	**0.02**
	**stearoylcarnitine**	**0.46**	**0.27**	**0.83**	**0.61**	**0.45**	**0.75**
	**O-propanoylcarnitine**	**−0.69**	**−0.32**	**−0.27**	**0.61**	**−0.54**	**0.73**
	arachidonate	0.51	−0.29	0.34	0.54	-	
	cholesterol	0.28	0.20	0.48	−2.68		
	choline	0.29	2.46	0.16	0.81		
	L-carnitine	−0.38	−0.35	−0.23	0.63		
	O-butanoylcarnitine	−0.60	−0.24	−0.40	0.61		
	chenodeoxycholic acid	−1.64	−1.04	−1.43	−1.68		
	(R)-3-hydroxybutanoate			1.50	0.47	0.77	0.95
	16-hydroxyhexadecanoic acid			0.45	0.46	0.56	0.81
	3-hydroxyisobutyrate			1.03	0.62	0.53	0.72
	3-methyl-2-oxobutyrate			0.30	0.61	0.29	0.69
	sphinganine-1-phosphate			1.32	0.46	0.79	0.04
	L-oleoylcarnitine			0.55	0.60	0.34	0.72
	mead acid			0.43	−0.36	0.78	−0.60
	tauroursodeoxycholate			0.55	−1.30	0.97	−1.96
**Amino Acid**	**methylimidazoleacetic acid**	**−1.02**	**−0.49**	**−3.64**	**0.45**	**−2.12**	**0.94**
	creatine	0.43	1.35	0.38	0.70		
	GSSG	−1.15	−0.60	−0.58	0.53		
	aspartate	−0.38	−0.49	−0.17	0.48		
	citrulline	−0.20	−0.67	−0.29	0.63		
	cysteine	−0.56	−0.53	−0.47	0.62		
	guanidinoacetate	−0.79	−0.39	−0.45	0.36		
	ornithine	−0.64	−0.79	−0.23	0.62		
	arginine	−0.27	0.94	−0.17	0.67		
	5-oxoproline			0.24	0.53	0.23	1.34
	glutamine			0.14	0.51	0.24	1.15
	serotonin			3.31	0.58	2.21	0.62
	urocanate			0.40	0.43	1.23	0.85
	kynurenine			−0.30	0.59	−0.45	0.80
**Carb**	fructose	0.53	0.36	0.56	0.55		
	D-glucitol			−2.47	0.61	−2.32	0.69
**Nucleo-tide**	**cytidine**	**0.99**	**1.18**	**1.14**	**0.38**	**0.64**	**−0.05**
	spermidine	1.10	0.82	0.96	0.14		
	uracil	0.51	0.42	0.41	0.55		
	thymidine			0.33	0.36	0.31	0.14
	urate			0.38	0.80	0.34	0.01
	uridine			0.10	0.26	0.46	0.12
	thymine			0.33	0.46	0.61	−0.30
**Cofactor**	**4-pyridoxate**	**0.39**	**−0.67**	**0.34**	**0.61**	**0.39**	**0.69**
	D-gluconic acid			1.16	0.33	0.57	0.51
	oxalate			0.23	0.18	0.43	0.40
	threonate			0.42	0.00	0.58	0.35

## Supplementary Materials

The following are available online at https://www.mdpi.com/1422-0067/21/21/8250/s1, Figure S1: Preliminary dose-response studies using clinical chemistry markers to determine toxicant dose and time points; Table S1: List of all genes in each study together with their significance values; Table S2: All the common genes across the three toxicants under the studied conditions; Table S3: Summary of changes in plasma metabolites for CCl4 study.
