# Supplementary material for: Toxicant-Induced Metabolic Alterations in Lipid and Amino Acid Pathways Are Predictive of Acute Liver Toxicity in Rats
Source: Int J Mol Sci. 2020 Nov 4;21(21):8250. doi: 10.3390/ijms21218250 (PMC7663358; doi:10.3390/ijms21218250)
Supplement: Supplementary file 1 [file ijms-21-08250-s001.zip › Supplementary Figure S1.docx]

**Supplementary Material:**

**Toxicant-induced metabolic alterations in lipid and amino acid pathways are predictive of acute liver toxicity in rats**

Venkat R. Pannala^1, 2, *^, Shanea K. Estes^3^, Mohsin Rahim^4^**,** Irina Trenary^4^, Tracy P. O’Brien^3^, Chiyo Shiota^3^, Richard L. Printz^3^, Jaques Reifman^1^, Masakazu Shiota^3^, Jamey D. Young^3, 4, *^ & Anders Wallqvist^1, *^

^1^Department of Defense Biotechnology High Performance Computing Software Applications Institute, Telemedicine and Advanced Technology Research Center, U.S. Army Medical Research and Development Command, Fort Detrick, MD 21702, USA

^2^The Henry M. Jackson Foundation for the Advancement of Military Medicine, Inc., Bethesda, MD 20817, USA

^3^Department of Molecular Physiology and Biophysics, Vanderbilt University School of Medicine, Nashville, TN 37232, USA

^4^Department of Chemical and Biomolecular Engineering, Vanderbilt University School of Engineering, Nashville, TN 37232, USA





**Figure S1:** Preliminary dose-response studies using clinical chemistry markers to determine toxicant dose and time points to measure perturbations in liver metabolism. Time course of levels of liver-injury markers of ALT and AST for acetaminophen (a and b) [1], for bromobenzene (c and d) [2], and for carbon tetrachloride (e and f) at different concentrations.

**References**

1. Pannala, V. R.; Wall, M. L.; Estes, S. K.; Trenary, I.; O'Brien, T. P.; Printz, R. L.; Vinnakota, K. C.; Reifman, J.; Shiota, M.; Young, J. D.; Wallqvist, A., Metabolic network-based predictions of toxicant-induced metabolite changes in the laboratory rat. *Sci Rep* **2018,** 8, (1), 11678.

2. Pannala, V. R.; Estes, S. K.; Rahim, M.; Trenary, I.; O'Brien, T. P.; Shiota, C.; Printz, R. L.; Reifman, J.; Oyama, T.; Shiota, M.; Young, J. D.; Wallqvist, A., Mechanism-based identification of plasma metabolites associated with liver toxicity. *Toxicology* **2020,** 441, 152493.
